# On the Diagnosis of Cancer and of Simple Dilatation of the Stomach

**Published:** 1895-09

**Authors:** J. Michell Clarke

**Affiliations:** Physician to the Bristol General Hospital, and Lecturer on Practical Physiology, University College, Bristol


### ON THE DIAGNOSIS OF CANCER AND OF SIMPLE DILATATION OF THE STOMACH.

BY

J. MICHELL CLARKE, M.A., M.D. Cantab., M.R.C.P. Lond.,

*Physician to the Bristol General Hospital, and Lecturer on Practical Physiology, University College, Bristol.*

---

IN the diagnosis and treatment of gastric disorders, the value of a systematic examination of the contents of the stomach at various stages after the ingestion of a test meal is now generally admitted. The large amount of work on this subject that has been done in the last few years has added greatly to the knowledge of the processes occurring during digestion, both in normal and in pathological states of the stomach. The earlier researches seemed to indicate that free HCl was absent from the gastric juice in cases of gastric cancer, and this absence of free HCl was held to be pathognomonic of cancer; but it has now been shown that HCl may be absent from the stomach in many other conditions, *e.g.* the acute fevers, chronic gastritis, and atrophy of the gastric mucous membrane. It is still true, however, that free HCl is far more frequently absent in cancer than in any other disease of the stomach. Without going fully into the literature, I may quote some recent observations by Schüle,[1] who found that in 88 cases of cancer of the stomach HCl was present in the gastric contents in 10, and sometimes absent,

[1] *München. med. Wchnschr.*, 1894, p. 737.

sometimes present, in 5 more; in the rest—83 per cent.—it was absent. In 21 cases of cancer examined by Ewald, HCl was absent in all. Schüle cites the results of the examination of 210 cases of non-malignant diseases of the stomach by Wagner: in 33, or only 15 per cent., HCl was absent. Further, when HCl is present in cases of cancer, it is in greatly reduced amount, on the average less than half of the normal. Salzer,[1] however, gives a higher percentage than most other observers for the absence of HCl in non-malignant stomach disorders. Out of 97 cases of chronic gastritis and nervous dyspepsia, HCl was absent in 35. In 4 cases of cancer, it was absent in all.

Since the work of Boas much attention has been paid to the question of the presence of lactic acid during digestion in cancer of the stomach. It is now considered that the only acid secreted by the gastric glands is hydrochloric, and that lactic acid arises from the fermentation of carbohydrates in the food. It is always present during the first 30–45 minutes of digestion after carbohydrates have been taken. Boas showed that all the test-meals contain lactic acid or its salts. Hence he gives simply oatmeal water gruel, and insists on the importance of a preliminary washing-out of the stomach. He also describes a more elaborate test for lactic acid than Uffelmann's reagent. Working in this way, he found lactic acid to be absent in normal digestion and generally in non-malignant disorders of the stomach, whilst it was constantly present in considerable quantity in 20 out of 21 cases of cancer. These results have been confirmed by others. The following statistics are taken from a valuable paper on "The Early Diagnosis of Cancer of the Stomach," by Dr. Morris Manges.[2] Cohnheim found lactic acid present in 9 out of 10 cases of gastric cancer, in 4 cases of œsophageal cancer; whilst it was absent in 6 cases of dilatation of the stomach, and in 14 cases of advanced chronic gastritis, in which HCl was also absent. Ewald found large quantities of lactic acid in 22 out of 24 cases of cancer of the stomach, and, on the other hand, Strauss found it present in only 5 out of 129 cases of non-malignant gastric diseases.

[1] *Johns Hopkins Hosp. Bull.*, vol. iv., 1893, p. 121.
[2] *Med. Rec.*, vol. xlvii., 1895, p. 518.

In a recent paper Boas[1] reaffirms his former statements, remarking that Uffelmann's test is not very sensitive, and that care must be taken to exclude lactic acid from the test meal. Dr. Manges, however, thinks that Uffelmann's test is sufficiently delicate for clinical purposes; other more elaborate tests take too much time for practical clinical work. The lactic acid may be taken up from the gastric juice by agitation with ether, and the latter evaporated, or a little of the ether extract directly added to the test. The general result of all researches on the gastric contents in cases of cancer of the stomach is to show that if in any case of gastric disorder HCl is found absent on several examinations at different intervals of time after a test meal, and at the same time lactic acid is present in considerable quantity; whilst there is stagnation of the stomach contents, the diagnosis is strongly in favour of cancer, even if there is no palpable tumour.

In the clinical examination of the gastric contents it is necessary that the tests employed should be fairly simple, easy of application, and not consume too much time in carrying out. Very useful information on the mode of procedure, mostly derived from the work of Martius and Lüttke, is given in Prof. Gamgee's *Text-book of the Physiological Chemistry of the Animal Body*.[2]

I may briefly recapitulate a useful method of work, taken chiefly from the above. The acidity is determined with phenolphthaleïn as an indicator, which, according to Martius and Lüttke, only indicates the acid reaction, and not the presence of free acid, which latter is determined by the use of Congo red papers. Congo red, however, will not show whether we have to do with mineral or organic acid. Gunzberg's reagent determines this, as it reacts only to a mineral acid. I have also used Benzo-purpurin papers, but find them a less delicate and certain test than Gunzberg's reagent. Uffelmann's reagent is used for lactic acid, preferably after extraction of the gastric juice with ether. Finally, the gastric contents are tested for peptones and sugar, and a little put aside in a warm place with some small cubes of hard-boiled white of egg to test its digestive powers.

[1] Abstract in *Centralbl. f. innere Med.*, 1895, p. 610.

[2] Vol. ii., 1893, p. 495, *et seq.*

I have also used Gunzberg's test as a quantitative one for HCl. Martius and Lüttke recommend that the gastric juice should be tested unfiltered.

It is important that the stomach should be emptied before the test meal is given. I have more than once found presence of lactic acid and absence of HCl on examination of a case of dilated stomach in which this had not been done, and in which the stomach contained stagnated fermenting material; whilst a subsequent examination after previous lavage showed the presence of HCl in normal quantity, and absence of lactic acid. In cases of dilatation of the stomach it is often interesting to note how the gastric juice improves in the quantity of HCl present, and in peptic power, during treatment by regular washing-out of the stomach and the administration of a proper diet. It is hardly necessary to say that more than one examination should always be carried out, and that no reliance can be placed on the examination of vomited matters. The cases examined, which were carefully investigated, were 23 in number; the number of examinations carried out was 62. I am indebted to my former clinical clerks, Mr. W. P. Taylor, Mr. J. W. Rose, and Mr. W. T. Lydall, for help with them.

Of these, 8 were cases of gastric cancer, in which the diagnosis was confirmed either by necropsy or by the appearance of a palpable tumour. In all HCl was constantly absent: lactic acid was present in 6, generally in considerable amount. This point was not investigated in 2 of the cases. In 10 cases of non-malignant dilatation of the stomach, HCl was present in all, lactic acid in 4 cases constantly, only occasionally in 1, and in 1 case it was not tested for. Except, however, in a few of the cases examined lately, special care was not taken to exclude carbohydrates and lactic acid from the test meal, so that the above results can only be taken to show that under such conditions lactic acid is more frequently present in cases of cancer than in non-malignant gastric affections. The other cases included one of a cancerous growth in the great omentum, not involving the stomach. In this case HCl was present, lactic acid absent. In a case of ulcer of the stomach in a girl the total acidity was 60, hydrochloric acid present in small amount, a faint reaction for lactic acid, and butyric acid present on three occasions, apparently in considerable amount.

It may be stated that in this paper, following the suggestion of Ewald, the total acidity of the gastric contents is expressed by the number of c.cm. of decinormal caustic soda solution requisite to neutralise 100 c.cm. of it. In a case of the common form of dyspepsia occurring in chlorosis, the acidity of the gastric contents was 20, 60, and 83. HCl was present on the first and third occasions, on the second it was absent. The tests were made one hour, 1½ hours, and two hours after food respectively. Dr. K. Osswald[1] has found that in dyspeptic troubles in chlorosis there is generally deficient HCl. As a matter of practical experience, I have found that only in a minority of cases is relief of the dyspeptic symptoms obtained by the administration of HCl after meals.

In one case of gastralgia HCl was persistently absent from the gastric contents. The patient was a man aged 53, who was admitted into the hospital on several occasions with constant nausea and intense pain in the epigastrium, relieved by vomiting, which took place soon after any food was taken. No dilatation of the stomach was ever made out. The pain was agonising in severity. The patient had been a heavy drinker. There was no evidence of ulcer of the stomach. The long duration of the case, and the fact that he has remained well for twelve months since his last stay in the hospital, preclude cancer. Lactic acid was found in the gastric contents once only, and, as said above, HCl was absent on all occasions, the examinations being made at varying times after a meal.

Returning to the cases of cancer, the average duration of the illness was about two years. Patients appear to come to the hospital late in the illness, as in several of them symptoms had been present for a year before admission. It is not common to find a history of long antecedent dyspepsia in cases of gastric cancer, but in one man there had been symptoms of dyspepsia for five or six years previous to the discovery of a tumour at the pylorus. In one case presenting the typical symptoms of cancer at the cardiac end of the stomach, the results of ex-

[1] *München. med. Wchnschr.*, 1894, p. 557.

amination of the gastric contents were of great aid in the diagnosis, as during life no tumour could be felt; at the necropsy a tumour the size of a large orange was found at the cardiac end, around the opening of the œsophagus.

In two cases of cancer, peritonitis occurred without perforation shortly before the fatal termination. I have not been able to find other instances of this complication of gastric cancer, which must be a rare one apart from perforation of a malignant ulcer. The first patient, a man aged 75, had been ill for two years with gastric symptoms, and on admission a rounded tumour was felt to the right of and just above the umbilicus; this was recognised as a pyloric tumour. He emaciated and grew slowly worse, but without obvious cause was one day seized with a rigor; his temperature went up to 102·6°, and he complained of great pain and tenderness over the tumour. He died the next day, and the necropsy disclosed a large mass of new growth at the pylorus. Over the growth the peritoneum was thickened and covered with a layer of lymph, the fat was indurated, and the liver and under surface of the diaphragm were coated with recent lymph. The inflammation was confined to these situations. No perforation was found, but the growth in one place was excessively thin, and here the most marked changes in the peritoneum occurred.

In a second case of pyloric cancer, in a collier aged 61, peritonitis without perforation also occurred, the peritonitis being here general, and there was also purulent effusion into the left pleural cavity.

Of the cases of dilatation of the stomach, 8 were men and 3 women. The ages varied from 18 to 60 years; in 6 it was between 45 and 60 years. With the possible exception of cases of chronic ulcer with much thickening, giving rise to a palpable tumour, no gastric disorder gives rise to greater difficulty of diagnosis from cancer than simple chronic dilatation of the stomach in elderly persons; and it is precisely in this disease that the examination of the gastric contents gives such valuable means of attaining an exact diagnosis. To take an instance, a woman aged 59, was admitted complaining of pain after food, constant nausea, vomiting after food, flatulence, constipation, and loss of flesh. She was pale and thin; the stomach was greatly distended, with areas of marked tenderness over the ensiform cartilage, in the right epigastrium, between the shoulders, and at the angle of the left scapula. The tender area over the vertex of the skull described by Dr. Head[1] as associated with stomach disorder was well marked, and pressure on this spot gave rise to pain in the epigastrium. The age, emaciation, and general symptoms strongly pointed to cancer of the stomach; but examination of the gastric contents invariably showed the presence of HCl in normal or excessive quantity, and absence of lactic acid. For a time she did not mend, but then began to improve slowly; and after three months' treatment she was cured, had put on flesh, and the stomach had returned to its normal size.

One patient aged 56, who had suffered from dyspepsia of twelve years' standing, due to a dilated stomach, had five years before undergone a novel cure. He was advised to take a sea-voyage, and was violently sea-sick for some days, and after this remained free from dyspepsia for fourteen months. The sea-sickness presumably acted by thoroughly emptying the stomach of its stagnating contents. He went out of the hospital quite free from symptoms after three weeks' treatment by washing out the stomach.

In a man aged 60, dilatation of the stomach was of the rare form produced by the cicatrisation of a healed ulcer in the region of the pylorus. Five years previous to his fatal illness he was under my care with well-marked symptoms of gastric dilatation, and obtained much relief from washing out the stomach. After leaving the hospital he procured a tube, and had been in the habit during the whole time of passing the tube and emptying his stomach three or four times every day. He had suffered from flatulence, great pain in the stomach, and vomiting after food for twenty years, the vomiting generally, but not always, relieving the pain. He had brought up a very large quantity of blood on two occasions, seven and nine years previously. He was re-admitted on Sept. 21, 1894, with the same dyspeptic symptoms as before, in a more aggravated form. He was pale and emaciated, heart and lungs were normal, the stomach was dis-

[1] *Brain*, vol. xvii., 1894, p. 339.

tended, and a splashing sound was easily obtained over it. The abdomen could be thoroughly explored, and no tumour was felt. There was marked myoidema in the pectoral muscles, and a "goose-skin reflex" at once appeared along the lines formed by drawing the finger-nail over any part of the abdomen. The epigastrium was marked transversely by eight or nine lineæ albicantes, due to frequent distension of the abdomen by the enormously dilated stomach. When distended the stomach seemed to occupy the whole of the left side of the abdomen, extending downwards almost to the pubes. The peculiar nodosities on the fingers described by Bouchard[1] as present in prolonged cases of dilated stomach were very well marked, much more so than I have ever seen in any previous instance, and consisted in an enlargement or nodosity of the base of the second phalanges at the first phalangeal joint, affecting all the fingers. Examination of the gastric contents: Total acidity, 100; HCl absent, or in faint traces $\frac{3}{4}$ hour after the test meal; at $1\frac{1}{2}$ and $2\frac{1}{2}$ hours after it, present in large quantity; lactic acid absent; gastric juice had active digestive properties.

The patient suffered much from sleeplessness and from obstinate constipation. He could not take milk in any form, peptonised or with lime and barley water, and indeed seemed very much worse and more uncomfortable when taking fluids. He was therefore put as far as possible on an absolutely dry diet of light digestible solids, with a tumbler of hot water night and morning. On this treatment, with lavage of the stomach every morning, he improved somewhat.

The history of the case and the symptoms pointed to a contracting ulcer as the cause of the dilatation. The question of dilating the pylorus was taken into consideration, but the patient died quite suddenly and unexpectedly during the night of Nov. 5th. At the necropsy the stomach was found to be very large, its coats much thickened, the mucous membrane a dull slate colour, coated with adherent mucus, with some ecchymotic patches along its lower border. About four inches from the pylorus on the posterior border was a healed ulcer the size of a threepenny piece, with some contraction of tissues around it.

[1] *Leçons sur les Auto-intoxications dans les Maladies.* Paris. 1887.

The pylorus barely admitted the little finger; its walls were thickened and tough ; on the gastric side was a linear scar (old ulcer) in the middle of a small area of atrophied mucous membrane. There was here some puckering of the tissues, which was also visible externally. There was no new growth.

In cases of dyspepsia where dilatation of the stomach with stagnation of its contents is suspected it is, of course, very important to determine the length of time during which the food remains in the stomach. The administration of salol with determination of the interval after which salicyluric acid appears in the urine by the iron reaction was the method adopted in my earlier cases, but later researches have thrown considerable doubt on the accuracy of this test for the motility of the stomach, so that it can no longer be considered as a trustworthy one ; and the modification of the test by taking the number of hours that elapse before salicyluric acid disappears from the urine does not seem any more satisfactory. Probably the best method is to pass a tube at an interval of time after a meal at which the stomach should have discharged its contents into the duodenum, and ascertain whether the viscus is empty. With regard to the etiology and treatment of non-malignant dilatation of the stomach, so much has been written of late on the subject that a few words will suffice. A history of long-continued dyspepsia gradually becoming worse is a marked feature in most cases. It is often also to be noted that the patient's muscular system is weak and flabby. It is probable that dilatation gradually ensues on repeated distension of the stomach from flatulent dyspepsia, its muscular walls being weak and atonic. In the treatment of the mildest cases of not long duration in young or middle-aged adults, a cure is often quickly effected by careful regulation of the diet, with mild aperients and ordinary dyspeptic remedies. In others a little more severe, this treatment should be preceded by a thorough washing-out of the stomach once or twice. One thorough emptying of the stomach is sometimes enough, with advice as to diet and the administration for a short time of a mixture containing hydrochloric acid and nux vomica, or subnitrate or subgallate of bismuth, to start the patient on the road to eupepsia. In the more severe cases, especially in elderly people with more or less general debility, rest in bed for a time is essential to successful treatment. The time during which this rest is necessary varies, and must depend on the progress made. As a rule, from two to three weeks in bed is necessary. In these cases great benefit is derived from lavage of the stomach once a day; but it is not always necessary, and often need not be continued long. There is rarely difficulty about it; the patients soon learn to swallow a soft tube, and the relief to pain and distension obtained, which is often immediate, makes them appreciate it. In this case, "*il n'y a que le premier pas qui coûte.*" Sometimes, as in the case mentioned above, they learn to pass a tube for themselves, and may have to be prevented from using it too frequently. The best time for lavage is the morning, before the food for the day is taken. For cleansing the stomach warm boracic acid solution gives good results. It is not advisable to pour in too much at a time or the distension of the stomach may be kept up; 1–1½ pints is sufficient. As to diet, it is best to begin with small quantities of milk at frequent intervals, peptonised, or with the addition of saccharated lime-water, then to increase the quantity given at a time, and to lengthen the intervals, and to gradually add other light and nutritious articles of food until the patient slowly gets back to a full diet. When this is the case, he should keep to three meals a day only, take very little fluid at meal times, and a tumbler of hot water or other hot drink before breakfast and at night. Hot drinks are useful throughout treatment, as they promote contractility of the stomach. Some patients, however, do not do well on fluids, and are better treated almost from the first with a dry diet. In any case it is necessary for the patient to keep to a strict diet for a long time, eating only easily-digested food that does not contain a large indestigible residue, and being careful not to overload the stomach.

With regard to drugs, I have found a mixture containing strychnine, dilute hydrochloric acid, and chloroform water the most useful. Ipecacuanha, in doses of gr. ½–gr. ij., as recommended by French authors,[1] night and morning, is probably of service, and for the bowels, which are nearly invariably con-

[1] A. Mathieu, *Gaz. des Hôp.*, Fév. 5, 1888.

stipated, enemata, or a dose of a powder, also recommended in the above paper, composed of equal parts of acid tartrate of potassium, precipitated sulphur, and magnesia, will be found very useful.

In some cases the subnitrate of bismuth, with small doses of salicylate of soda, taken every three or four hours, gives more relief by checking abnormal fermentative processes. Faradisation of the stomach, which has been recommended, has not proved of much value in my hands. Most importance is to be given to rest, judicious regulation and variation of diet, with lavage in suitable cases, and the administration of suitable medicines for the relief of dyspeptic symptoms and of constipation. The result attained by these methods of treatment is generally very satisfactory.

---

